# Hostile attribution bias and angry rumination: A longitudinal study of undergraduate students

**DOI:** 10.1371/journal.pone.0217759

**Published:** 2019-05-31

**Authors:** Yueyue Wang, Shen Cao, Yan Dong, Ling-Xiang Xia

**Affiliations:** 1 Research Center of Psychology and Social Development, Southwest University, Chongqing, China; 2 Department of Psychology, Renmin University of China, Beijing, China; 3 Laboratory of Department of Psychology, Renmin University of China, Beijing, China; University of Lleida, SPAIN

## Abstract

Angry rumination and hostile attribution bias are important cognitive factors of aggression. Although prior theoretical models of aggression suggest that aggressive cognitive factors may influence each other, there are no studies examining the longitudinal relationship between angry rumination and hostile attribution bias. The present study used cross-lagged structural equation modeling to explore the longitudinal mutual relationship between hostile attribution bias and angry rumination; 941 undergraduate students (38.5% male) completed questionnaires assessing the variables at two time points. The results indicate that hostile attribution bias showed a small but statistically significant effect on angry rumination 6 months later, and angry rumination showed a quite small but marginally significant effect on hostile attribution bias across time. The present study supports the idea that hostile attribution bias influences angry rumination, and argue that the relationship between angry rumination and hostile attribution bias may be mutual. Additionally, the results suggest that there may be a causal relation of different aggression-related cognitive factors.

## Introduction

Angry rumination is prolonged thinking about personally meaningful angry events and is accompanied by angry feelings or thoughts about revenge [1, 2]. Angry rumination is also a pattern of thinking that specifically intensifies anger and increases aggressive tendencies [3]. Previous behavioral studies have shown that angry rumination is associated with negative outcomes, such as aggressive behavior [4], negative emotion, and depressive symptoms [5]. Furthermore, previous studies further revealed that angry rumination is one of the several important aggression-related cognitive factors [6]. In sum, angry rumination is a negative mental factor that should be subjected to intervention and changed. Thus, understanding the mental mechanisms of the development of angry rumination in daily life is both important and necessary. Recently, researchers have focused on the influencing factors in angry rumination, such as executive control [2], self-control [4], and self-compassion [7]. However, the aggression-related cognitive mechanism of the development of angry rumination in daily life is still unclear. Thus, we first aimed to discover one of the aggressive cognitive mechanisms that influence the frequency of angry rumination in daily life.

For the following reasons, we infer that the frequency of angry rumination in daily life is impacted by hostile attribution bias that makes negative content more accessible. First, the Social Information Processing (SIP) model emphasized the cycle of cognitive processes for aggression and suggested that aggressive cognitive factors may impact each other [8]. Furthermore, the Integrative Cognitive Model (ICM) of trait anger and reactive aggression suggested the potential cognitive processing approaches that hostile interpretations quite naturally lead to rumination upon such information [9]. In addition, negative interpretations of an ambiguous event can exacerbate the frequency to ruminate by fueling future thoughts through its disambiguated meaning; in this way, interpretation biases can contribute to the spiraling relation between rumination and negative mood states [10]. Hostile attribution bias is a kind of interpretation bias in which individuals are more likely to interpret ambiguous situations as hostile than benign [11]. Presumably, hostile attribution bias should impact angry rumination. Second, hostile attribution bias could facilitate the preference to memorize and recall angry information and feeling [9, 11]; moreover, hostile interpretation bias usually automatically captures attention [12, 13], thus hostile attribution bias may make an individual habitually focus on angry events or information. The prolonged allocating of attention to angry events is the key feature of angry rumination [14]. As such, hostile attribution bias may facilitate the development of the preference of angry rumination. Third, individuals who make hostile attribution bias usually tend to activate and adopt hostile schemas and scripts stored in long memory. Hostile schemas include memorized angry information derived from prior angry events, recalled when angry cues emerge in daily life [11]. Thus, hostile attribution bias naturally leads to increasing the frequency of ruminating on such angry information through hostile schemas [15]. Fourth, angry rumination refers to not only focusing and dwelling on angry moods but also analyzing the event’s causes and meaning [14, 16]. Hostile attribution bias emerges in ambiguous contexts [11, 17] when individuals are unsure of others’ intentions in daily life. The ambiguity of the intentions and the uncertainty of their own judgments would induce individuals with high hostile attribution bias to ruminate on the causes of angry events in daily life. The ruminations will repeat and continue even after the event is over to further prove the right of the attributions he/she made, which is exactly one of the key features of angry rumination[1]. Repeated thinking about the causes of angry events leads to more frequent angry rumination in daily life. In other words, individuals who have a tendency to make hostile attribution relative to ambiguous cues would likely ruminate on prior provoking events with anger in daily life.

On the other hand, the frequency of angry rumination in daily life seems to influence the development of hostile attribution bias. First, angry rumination involves understanding of causes [14], which may be the reason why angry rumination in daily life leads to the development of hostile attribution bias. Second, according to the daily experience, the anger-related cognition and emotion that has triggered angry rumination may make it easier for an individual to regard the ambiguous cue or unintentional provocation as hostile or intentional; similarly, the angry rumination may facilitate the development of hostile attribution bias in daily life. For example, a recent study found that a component of rumination leads to a negatively biased direction in interpreting ambiguous cues [18]. Thus, another aim of this study is exploring the longitudinal effect of angry rumination on hostile attribution bias at the same time.

In sum, most current studies [19, 20] explore the contribution of hostile attribution bias or angry rumination to aggression, and these studies explore the cross-sectional relationship between hostile attribution bias and angry rumination, but the longitudinal relationship between hostile attribution bias and angry rumination has not yet been tested directly. It is necessary and important to explore the predictive relationship between hostile attribution bias and angry rumination. At the theoretical level, this exploration may support and further clarify the cognitive theory of aggression. For example, it may support and improve aggressive models such as the SIP model and the General Aggression Model (GAM), which don’t elaborate on the role of angry rumination in aggressive cognition. At the practical level, this work may provide some theoretical basis and reference for correcting aggressive cognitions.

Based on the above-mentioned analyses, the goal of the present study was to test the mutual longitudinal relationship between hostile attribution bias and angry rumination. Specifically, we hypothesized that hostile attribution bias had longitudinal effects on the frequency of angry rumination 6 months later, and the frequency of angry rumination predicted hostile attribution bias over time.

## Methods

### Participants and procedure

The present investigation was a part of a large series surveying our research group. First, 1120 Chinese undergraduates were asked to complete the Angry Rumination Scale and Social Information Processing–Attribution Bias Questionnaire. Ten percent of the undergraduate students dropped out after Time 1 and 66 participants were excluded, resulting in 941 valid data sets in the 6-month interval (Time 1 –Time 2). The invalid data were excluded according to three criteria: 1) the subject did not participate in the second longitudinal survey; 2) the subject reported that he/she did not fill out the questionnaire seriously in the last item; and 3) the subject did not fill out 30% of the all items in the set of questionnaires. Participants were 18–27 years old (*M* = 21.09 years, *SD* = 1.07 years, 38.5% male). Data collection was conducted by trained research assistants. Prior to the survey, verbal informed consent was obtained. Then, the research assistants distributed the questionnaires to each participant. All participants completed the paper-and-pencil surveys in person. Participants received a payment of 10 CHY afterwards. Written informed consent was obtained from all students prior to the survey, and this research was approved by the Ethics Committee at the Faculty of Psychology, Southwest University, China.

### Measures

#### Hostile attribution bias

Hostile attribution bias was measured using the Hostile Attribution Bias subscale of the Social Information Processing–Attribution Bias Questionnaire (SIP–ABQ) [21], which included eight scenarios. Each scenario depicted a situation with a negative outcome, and the intentions of the character were ambiguous. Participants were asked to rate items, per scenario, on a scale ranging from 0 (not at all likely) to 3 (very likely). The sum of the 16 hostile interpretation item scores was considered the hostile attribution bias score. In the study, we utilized the Chinese version of the Hostile Attribution Bias subscale. We conducted a confirmatory factor analysis (CFA) to test the validity of the subscale in the current sample (*N* = 941). The CFA showed that the one factor model fit with the data well, RMSEA = 0.03, CFI = 0.999, TLI = 0.997, SRMR = 0.006. These results revealed that the Chinese version of the Hostile Attribution Bias subscale had good construct validity. In addition, the test-retest reliability was 0.61 (6-month interval) in the present sample. Cronbach’s alpha in the current sample was 0.88 at Time 1 and 0.91 at Time 2.

#### Angry rumination

The Angry Rumination Scale contains 19 items measuring the frequency to ruminate on angry events in daily life [14]. Participants were asked to rate the items on a 4-point scale (1 = almost never; 2 = sometimes; 3 = often; 4 = almost always). We utilized the Chinese version of the scale, which had good reliability and construct validity [22]. We conducted the confirmatory factor analysis (CFA) to test the construct validity of it in the present sample. The results showed adequate fit to the data, *χ*^2^ = 539.49, *df* = 146, RMSEA = 0.05, CFI = 0.94, TLI = 0.92, SRMR = 0.05. In addition, the Cronbach’s alpha in the current sample was 0.94 at Time 1 and 0.93 at Time 2, and the test-retest reliability was 0.63 (6-month interval). These results revealed that the reliability and validity of the Chinese version of the Angry Rumination Scale was adequate.

## Data analyses

Descriptive statistics and correlation analysis were performed using SPSS 22.0 software. Then, confirmatory factor analyses (CFAs) and cross-lagged model analysis were conducted with Mplus 7.0. Latent Variable Structural Equation Modeling was used in the present research because, compared with the manifest variable model, it has several advantages, such as the ability to take measurement error into account, involving whole systems of conceptual relationships, and the potential to improve scale development in the field by providing statistical tests of construct dimensionality [23]. In the present study, all variables in the model at Time 1 and Time 2 were latent variables. The latent variables were created using parcels of items from scales, because there are several strengths of item parceling: increase the stability of the parameter estimates; improve the variable-to-sample-size ratio; help mitigate the problem of non-normality; enhance the communality; increase the common-to-unique ratio for each indicator; and reduce random error [24]. In order to balance the load of items, the items of hostile attribution bias and angry rumination were assigned to four parcels by item-to-construct balance method [25], respectively. For hostile attribution bias, the original 16 items were replaced by 4 parcels of 4 items each; for angry rumination, the 19 items were replaced by 3 parcels of 5 items and 1 parcel of 4 items. Then, we tested the model fit of the measurement model of hostile attribution bias and angry rumination at the two time points, respectively. Finally, we conducted the longitudinal cross-lagged panel analysis between the hostile attribution bias and angry rumination.

We employed four widely used model fit indices: the comparative fit index (CFI) and Tucker–Lewis index (TLI), with values higher than .95 suggesting an excellent fit; root-mean-square error of approximation (RMSEA), with 90% confidence interval (CI) and standardized root-mean-square residual (SRMR), with values less than .05 suggesting a good fit [26, 27].

Concerning the missing data, full information maximum likelihood (FIML) estimation was adopted to handle the missing data. FIML estimation is an efficient and unbiased method that uses all available information to estimate parameters when data are missing [28].

## Results

### Descriptive statistics

The means, standard deviations, and correlations of variables are presented in Table 1. The results indicated that angry rumination and hostile attribution bias were significantly and positively associated with one another at each time point.

**Table 1:** Means (M), Standard Deviations (SD), and correlations between hostile attribution bias and angry rumination in sample of chinese undergraduate students.

Variables	*M*	*SD*	1	2	3
1. AR1	2.97	1.26	1		
2. ABQ1	1.09	0.43	0.36**	1	
3. AR2	3.01	1.27	0.62**	0.31**	1
4. ABQ2	1.12	0.44	0.28**	0.61**	0.35**

*Note*. ABQ = hostile attribution bias; AR = angry rumination

**p < 0.01

### The cross-lagged path model

First, the measurement models in Time 1 and Time 2, which included latent variables for hostile attribution bias and angry rumination, were tested, respectively. All model fit indices of the two measurement models showed that the latent model fit the data well: *χ*^2^ = 32.49–60.43, *df* = 19, *p* < 0.001, CFI = 0.993–0.998, TLI = 0.990–0.997, RMSEA = 0.02–0.04 (90% CI), SRMR = 0.016–0.19.

**Fig 1:**
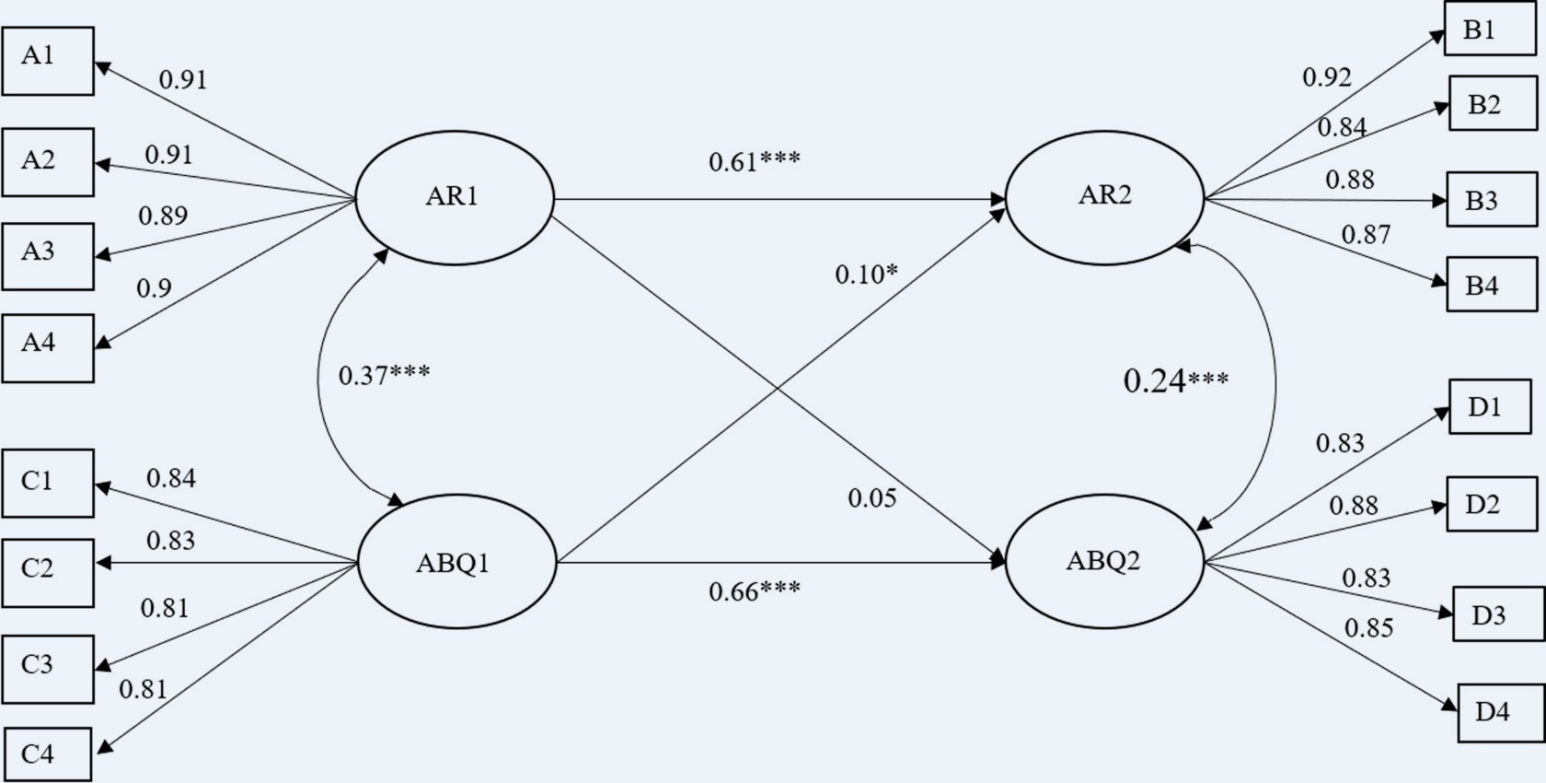
Standardized structural model of longitudinal cross-lagged panel analysis on hostile attribution bias and angry rumination across two waves. Note, ABQ = hostile attribution; AR = angry rumination; a1 = angry rumination parcel 1 of time 1; a2 = angry rumination parcel 2 of time 1; a3 = angry rumination parcel 3 of time 1; a4 = angry rumination parcel 4 of time 1; b1 = angry rumination parcel 1 of time 2; b2 = angry rumination parcel 2 of time 2; b3 = angry rumination parcel 3 of time 2; b4 = angry rumination parcel 4 of time 2; c1 = hostile attribution bias parcel 1 of time1; c2 = hostile attribution bias parcel 2 of time1; c3 = hostile attribution bias parcel 3 of time1; c4 = hostile attribution bias parcel 4 of time1; d1 = hostile attribution bias parcel 1 of time2; d2 = hostile attribution bias parcel 2 of time2; d3 = hostile attribution bias parcel 3 of time2; d4 = hostile attribution bias parcel 4 of time2. All the reported parameters are standardized. ***p < 0.001.

Then, longitudinal cross-lagged panel analysis was conducted and the results are presented in Fig 1. The fit indices indicated that the final model fit the data well: *χ*^2^(99) = 201.56, *df* = 98, *p* < 0.001, CFI = 0.99, TLI = 0.99, RMSEA = 0.03 (90% CI ranged from 0.02 to 0.04), SRMR = 0.02. Hostile attribution bias at Time 1 was found to have cross-lagged effects on Time 2 angry rumination (β = 0.1, *p* = 0.008). The cross-lagged effect of angry rumination at Time 1 on hostile attribution bias at Time 2 seemed to be marginally significant (β *=* 0.05, *p =* 0.06).

## Discussion

### Longitudinal relationship between hostile attribution bias and angry rumination

In this study, we used the cross-lagged analyses to explore the longitudinal relationships between hostile attribution bias and angry rumination in healthy individuals. The results indicated that hostile attribution bias showed a small but statistically positive effect on the frequency of angry rumination 6 months later. Additionally, although the longitudinal effect of angry rumination on hostile attribution bias was quite small, it seems to be marginally significant. These results suggest that, on the one hand, hostile attribution bias definitely had longitudinal effect on angry rumination and angry rumination may also have longitudinal effect on hostile attribution bias; on the other hand, the effects seem to be small and should not be overestimated. In other words, the present results should use cautiously.

As mentioned above, there are two reasons can explain why hostile attribution bias can predict the frequency of angry rumination. First, hostile attribution bias can exacerbate the frequency of rumination on angry events [10, 29]. Second, hostile attribution bias and angry rumination both refer to thinking about the reasons of the provoking event, and the ambiguity of the situation and uncertainty of judgment make individuals more easily form hostile attributions and habitually ruminate on the reasons for the angry events [16].

These findings lead to interesting suggestions that hostile attribution bias in angry events may lead to relatively more frequent angry rumination after these events. In other words, the speculation on others’ hostile intentions in angry events may transform into angry rumination to some degree. Thus, the hostile intention analyzing may be one of the sources of angry rumination.

Although the longitudinal effect of angry rumination on hostile attribution bias was marginally significant and the effect was small, it suggested that, to a certain extent, angry rumination can longitudinally predict hostile attribution bias. This finding was partly consistent with previous studies showing that such ruminative thoughts fuel biased negative interpretations [30]. On the other hand, the question of whether the longitudinal effect of angry rumination on existing hostile attribution bias warrants further exploration. There are several reasons that may explain the possible longitudinal effect of angry rumination on hostile attribution bias. First, angry ruminators tend to repeatedly process an anger-inducing event in ways that make negative information salient. In this case, they may tend to find negative meaning in ambiguous situations and then adopt hostile attribution in everyday life [31]. Second, angry rumination constitutes prolonged post-event processing, and this process may strengthen an individual’s negative relation schema, thereby fueling one’s hostile attribution in ambiguous social situations [32].

### Limitations and future directions

There are several limitations to the present study. First, the present study took place over a short time span of 6 months. Although this length of time is worthy of study, it would be more informative to employ longer time periods to determine if stronger relationships are found. Second, when the subjects answered this questionnaire, they did not have a specific time frame (for example, the past six months), but based their answers on their past daily life experiences. While this may not have much impact on the results, future studies should be measured based on specific longitudinal intervals. Third, the current study represents the first step in exploring the relationship between hostile attribution bias and angry rumination by employing a longitudinal study using latent variable modeling. The results should be replicated by further studies and the mechanisms of this relationship should be explored. Fourth, we focused on the relationship between hostile attribution bias and angry rumination in general; future studies can further explore the relationship between hostile attribution bias and a specific dimension of angry rumination, such as analytical rumination, which involves determining why an event occurred by analyzing the event’s consequences [33].

### Contributions and implications

The present study may make some unique contributions. First, our results provide us with a new perspective on understanding the cognitive mechanism of aggression-related factors and help us develop a cognitive theory of aggression. Both hostile attribution bias and angry rumination are important cognitive factors in aggressive behavior [4, 34]. However, the longitudinal or causal relationship between them has been ignored in previous literature. To a certain extent, our results suggest that the longitudinal relationship between hostile attribution bias and angry rumination is mutual. This suggests that some aggression-related cognitive factors may influence others to some degree, and it is worth exploring the relation among aggression-related cognitive factors in order to better understand the cognitive mechanism of aggression further. On the one hand, the study, in part, supported the cognitive theories of aggression, such as the SIP and GAM models. On the other hand, the study may provide kinds of possibilities for further study by integrating these aggression-related cognitive factors and facilitate our further understanding of how aggression-related cognitive factors being linked, activated, and maintained in some extent. Second, our study has focused the relationship from between interpretation bias and rumination to between hostile attribution bias and angry rumination, partly supporting the basic idea that interpretation bias influences rumination. Third, existing research on the influencing factors of angry rumination has been mainly focused on self-related factors such as self-control or self-compassion [4, 7] and previous studies mostly focused on the state of angry rumination in experimental studies. Our results enrich the understanding of the development of angry rumination in some extent. Fourth, the present results suggest that hostile attribution bias may impact other aggression-related cognitive factors to some degree and may play a role in the development of aggressive cognition in daily life.

The current study has several implications for research and practice. First, it has potential implications for our understanding of the cognitive process involved in moving from anger to aggressive behavior. The ICM highlighted that the potential cognitive processing approaches (hostile interpretations, ruminative attention, and effortful control) are associated with anger and reactive aggression [9]. Our research further suggested the potentially predictable relationship between hostile attribution bias and angry rumination and offered a new possibility for future investigations regarding the cognitive bias of trait anger. Second, findings of this study also had some practical implications. For example, since hostile attribution bias, to some extent, fuels angry rumination, reducing the generation of hostile attribution bias may be a potential method to prevent angry rumination for psychological consultants. Specifically, when students’ information processing and thinking is negative or full of hostile perceiving, they should be guided to examine their attributions from different perspectives to reduce hostile perceiving and then avoid entering angry rumination phase to some degree.

## Supporting information

S1 Dataset Longitudinal hostile attribution bias and angry rumination. (SAV)
